# New record of parasitic protozoan and helminths in buffaloes from Paraguay

**DOI:** 10.5455/javar.2024.k846

**Published:** 2024-12-27

**Authors:** Griselda Meza Ocampos, Jorge Miret Riquelme

**Affiliations:** 1 Laboratorio de Biotecnología, Centro Multidisciplinario de Investigaciones Tecnológicas, Universidad Nacional de Asunción (CEMIT, UNA), San Lorenzo, Paraguay; 2 Instituto de Investigaciones en Ciencias de la Salud (ICCS). Universidad Nacional de Asunción (UNA), Centro Multidisciplinario de Investigaciones Tecnológicas (CEMIT), Campus Universitario San Lorenzo, San Lorenzo, Paraguay

**Keywords:** Coccidiosis, *Eimeria* spp., *Haemonchus* spp., *Moniezia* spp., Natural resistance

## Abstract

**Objective:**

This study aimed to evaluate the prevalence, abundance, and identification of genera of gastrointestinal parasites in buffaloes belonging to an establishment in Villa Oliva, Ñeembucú Department, Paraguay.

**Materials and Methods:**

A total of 117 buffaloes were included in the study and divided into three groups of 39 (n = 39) animals—Group 1: = <12 months (G1); Group 2: = <36 months (G2); and Group 3: >37 months (G3). All samples were tested using the saturated salt flotation. Eggs and oocyte counts were determined using McMaster’s method. Recuperation of larvae 3 was carried out after coproculture using the Baerman technique. Identification was based on morphological identification keys.

**Results:**

The presence of eggs and/or oocysts of parasites belonging to Cestoda, Protozoa, and Nematoda was noted. The prevalence of gastrointestinal nematode (GIN) was 36.75%. The highest abundance was observed in G1. After coproculture *Moniezia expanza*, *Eimeria* spp., and *Haemonchus* spp.; *Teladorsagia* spp./*Ostertagia* spp. were also identified. It is also observed that the incidence of nematode parasite infestation in female buffalo is high compared to males; however, in terms of microparasites, it is the opposite. According to our results, as buffalo age increased, parasite loads decreased considerably.

**Conclusion:**

Those results may link factors between hosts and the environment with the ability to maintain gastrointestinal infestation at levels that do not compromise health and body conditions. This study presented results of the prevalence, abundance, and identification of GINs from buffalos of Paraguay for the first time.

## Introduction

Buffaloes (*Bubalus bubalis*) are popularly known as black gold in South Asia. There are approximately 2,00,000,000 buffaloes in the world, mostly concentrated in India, Pakistan, and China, with 97% of the world’s population [1]. In America, buffaloes are concentrated in Brazil with more than 3.5 million and Venezuela with 2.1 million. In Paraguay, 15,000 heads of buffalo were reported [2].

Interactions between coccidia (microparasites), cestodes, and nematodes (macroparasites) have been well documented in buffaloes; these co-infections of gastrointestinal parasites cause serious economic losses in ruminants due to reduced milk production, low fertility, decreased working capacity, the cost of treatment, reduced body weight gain in young animals, and also increased susceptibility to other diseases [3,4,5].

Buffaloes have shown an excellent ability to adapt to the American tropics because they originate from tropical and subtropical areas of western Asia and have been selected naturally for their rusticity and adaptation to a medium of extreme marginality [6]. However, warm and humid climatic conditions in these areas are favorable for the development, survival, and translation of parasite-free life stages [7]. Knowledge of a specific pathogen is essential for establishing control measures for any disease [8,9].

Epidemiological knowledge of parasitic infections is very important to adopt effective control measures that reduce economic losses in animals [9] since several risk factors, such as health status, age, and sex, influence the prevalence of gastrointestinal parasites in animals. The mortality of the young is considered one of the main causes of losses in buffalo cattle production. Young animals’ mortality ranges from 7.1% to 17.9%, especially in the year of life [10].

The present study aimed to record the presence of gastrointestinal parasites in buffaloes for the first time in Paraguay. The data will help to understand the importance of gastrointestinal parasites for buffaloes and to learn about this alternative species to produce high-quality meat adapted to this country where the prevalence of gastrointestinal nematodes is high in cattle on natural pasture.

## Materials and Methods

### Ethical approval

All ethical concerns were considered during fecal sample collection from the study animals. For further compliance with ethical standards, mutual consent was made between the animal owner and investigators by briefing the study’s objectives. Stool collection is a part of routine veterinary procedures without any traumatic method. The work protocol was approved by the Scientific Committee of CEMIT.

### Area of study

The buffalo belongs to the estancia San Luis, located in the town of Villa Oliva, Department of Ñeembucú, Paraguay (25°98 S, 57°81°01N). The animals correspond to the so-called water buffalo (*B. bubalis*), mestizos of the Mediterranean, and Murrah breeds.

### Sample selection and collection

The buffaloes are divided and identified by category by age. Each of the sampled animals was identified by a unique code, and data regarding age and sex were recorded. A total of 117 animals were included randomly in the study and divided into three groups of 39 (*n =* 39) animals, as detailed in the following—Group 1: = <12 months (male/female) (G1); Group 2: = <36 months (male/female), with identification (G2); and Group 3: = >37 months (G3). The animals in this establishment were not subjected to a deworming or antiparasitic scheme after, during, or before the study. The management system is free grazing in wet and dry areas. The fecal samples were taken, while the buffaloes were immobilized in the cattle crushes. Fecal samples were taken directly from the rectum with a palpation sleeve previously moistened with Vaseline®. The samples were stored in the same plastic sleeve, tying them, previously removing most of the air, to later be stored in an isopored container with ice to avoid the development of the eggs during transport [3,11].

### Sample preparation for parasitological screening

Fecal samples were examined utilizing established parasitological screening methods for intestinal parasites, specifically the simple salt flotation procedure succeeded by sedimentation and direct saline observations. All animals exhibiting 50 eggs per gram (EPG) and/or 50 oocysts per gram (OPG), corresponding to the microscopic identification of a strongyle egg or a coccidia oocyst, respectively, were deemed positive for parasitic infection [12–14].

### Morphological identification and quantification

The parasite eggs/oocysts, larvae, and cysts were analyzed and classified to the genus level using microscopy, following morphological identification keys [15,16]. The modified McMaster technique was employed for quantitative analysis to quantify eggs/OPG of feces (EPG/OPG) [14]. All samples were subsequently processed using the Baermann approach by placing the larval culture immediately into conical sedimentation flasks filled with water and allowing it to sit for 12 h [17].

### Calculation of prevalence and abundance

Prevalence (positive number/total sampled) and abundance (egg or oocyst count/gram feces) values were calculated according to age and sex and expressed in percentage [18–21]. Abundance is the arithmetic mean of the parasite load [22].

### Index of importance of the number EPG/OPG

The importance of the number of EPG/OPG was based on the following classification—negative: (0 EPG/OPG), mild infection: (<200 EPG/OPG), moderate infection: (<700 EPG/OPG), and high infection: (>700 EPG/OPG) considered accumulators of parasites [14].

### Statistical analysis

Data were analyzed for differences among groups (G1–G3) and males and females using one-way ANOVA, with a significant level (*p*-value) reported. *p* < 0.05 Turkey examination. Statistical analyses were conducted using the Prisma® software. Generalized linear models were employed to identify the primary predictors of parasite infections, with model selection conducted on the dependent variables: egg counts and oocyst counts per class. Co-infecting parasite loads were modeled as logarithms (fecal egg count +1) for Nematoda and logarithms (oocyte count +1) for Protozoa, serving as independent variables. Host age was incorporated into the models as a categorical variable (1, 2, and 3) due to its superior fit, which denotes the category as a linear predictor.

## Results and Discussion

### Prevalence and index of importance of parasites in buffaloes

Throughout the study period, 117 fecal samples from the buffalo herd were analyzed, revealing that 43 samples were infected with one or more species of gastrointestinal parasites, resulting in an overall prevalence of 36.75%. The eggs and larvae were enumerated and classified by phylum as nematodes, cestodes, and protozoa. The overall prevalence was 22%, 12%, and 3% for Nematoda, Cestoda, and Protozoa, respectively. Table 1 illustrates the prevalence and significance of gastrointestinal parasitism in the sampled animals.

The assessment of parasitic infestation must be conducted comprehensively, as it relies on the interplay among the parasite, host animal, and pasture. The reduced levels lead to the expulsion of eggs and/or oocysts in the Nematoda, Cestoda, and Protozoa phyla, substantiating the concept of inherent resistance in buffaloes relative to bovines [22]. Due to the low egg count in animals under 1 year of age and the lack of eggs in a significant proportion (<700EPG) of samples from adult animals.

**Figure 1.**
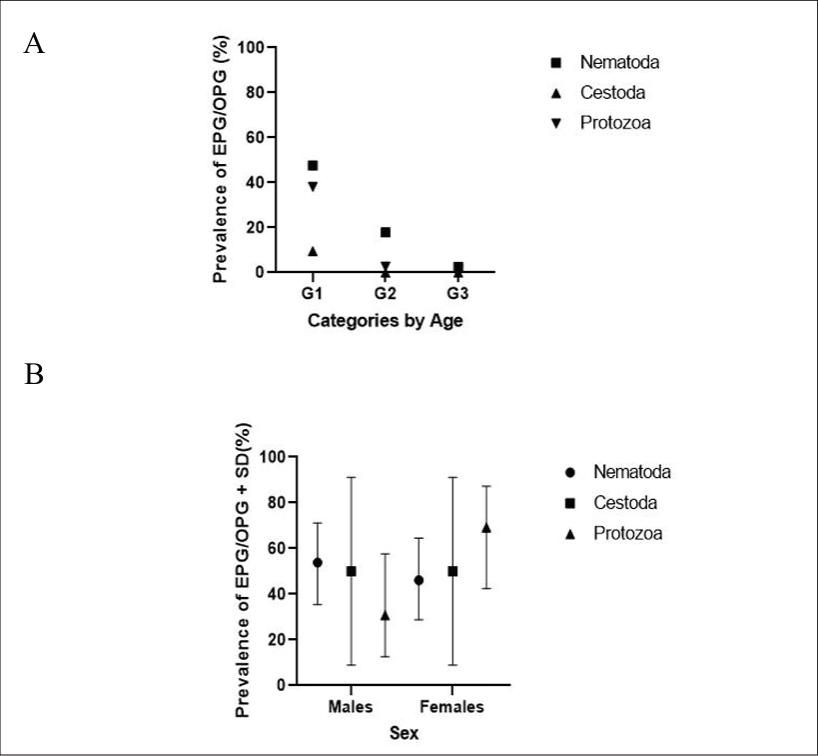
Mean prevalence by group and sex in buffaloes G1 had the highest prevalence (A, 47.5%) compared to G2 and G3 (sample size for each group: N = 39). G1 had the highest prevalence (A) 47.5% compared to G2 and G3 (sample size for each group: N = 39). (B) Females had a lower estimated prevalence compared to buffalo males in Nematoda (46% ± 53%), while in the Protozoa class, the females had 69% compared to 30% of males (B). *Indicates significant differences at p < 0.05.

The prevalence of both nematodes and coccidia was lower in older age categories and differed by sex (*p* > 0.05) (Fig. 1). The lower prevalence and HPG/OPG values obtained in adult animals are related, among other things, to the development of immunity toward parasites by age (G3 2.3, 0, and 2.3 in Nematoda, Cestoda, and Protozoa, respectively). Some of the animals in G3 were pregnant at the time of the study. The low prevalence of infestation in females could be explained by the stimulatory effect of estrogens on the immune system. This suggests a protective hormonal effect, whereas, in males, testosterone suppresses such a response [5,23–26].

### Abundance of gastrointestinal parasite infection

**Figure 2.**
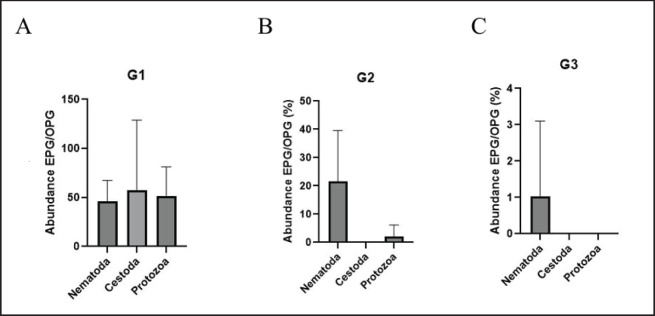
Abundance expressed in mean and SD of EPG/OPG by Groups G1, G2, and G3, and by Class (Nematoda, Cestoda, and Protozoa).

The abundance of infection was measured in terms of EPG/OPG by phylum. The Nematoda has an egg count ranging from 0 to 240 eggs/gm. The protozoa had an oocyte count that ranged from 0 to 240 oocysts/gram, and the Cestoda ranged from 0 to 1200 eggs/g. The abundance and importance of gastrointestinal parasitism in the sampled animals are shown in Figure 1, Table 1, Figure 2 and Table 2.

The highest abundance was observed in G1 in the Cestoda (57.00 ± 220.01 (*p* > 0.05). G1 presented differences in eggs and oocyte excretion with G2 and G3 (*p* < 0.05). Parasite abundance was also higher in calves and juveniles compared to the adults (G2 and G3).

### Identification of parasites and coinfections

**Figure 3.**
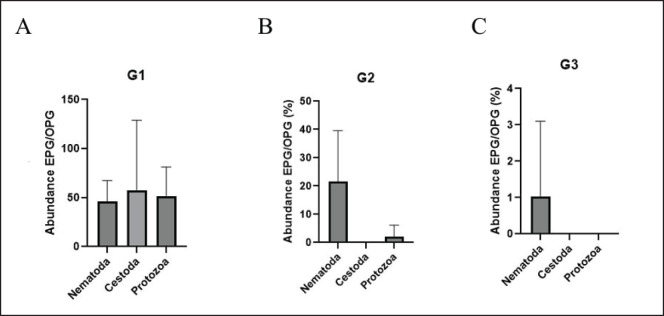
Prevalence of co-infected samples by Nematoda, Cestoda, and Protozoa.

The macroparasite species identified after coproculture were three genera of nematodes: *Haemonchus* spp., *Ostertagia* spp., and *Teladorsagia* spp. In addition, the cestode *M. expanza* has been identified. Oocytes of *Eimeria* spp. were identified as microparasites. In total, eight animals presented coinfection, of which the Nematoda–Protozoa coinfection was seven of eight animals and Nematoda–Cestoda one of eight, as observed in Figure 3.

Because nematode egg counts also reflect differences in parasite fecundity, variation in worm fecundity decreased with coinfection [5], and microparasites and nematodes were positively associated with infection abundance analyses. Increased Nematoda egg counts in young buffaloes, but not in adults, where it was associated with increased odds of coccidia infection.

The abundance of microparasites is higher in buffaloes co-infected with nematodes. We can also observe that, in G3, where the infestation rate is low or even null, we had a population of pregnant females. This low infestation rate is possible because, in females, estrogens increase the capacity of the monocyte–macrophage system to phagocytose antigenic particles and increase humoral immunity, while male hormones tend to suppress the humoral and cellular response [27].

In combination, the release of parasites related to the condition of host immune control and hypobiosis can give rise to a large number of infectious stages in the environment during a period when many hosts are less capable of defending themselves against invasion by parasites, leading to a strong seasonal signal in the parasite, which could coincide with the peripartum and high mortality rate in the first year of life.

The abundance in the class Nematoda was higher in the female buffalo compared to the male buffalo, but the nematode egg count did not vary between sexes. On the contrary, the abundance of coccidia was higher in male buffaloes. We should note that these results may also have been influenced by variations in worm fecundity because our analyses were based on fecal egg counts rather than parasite load [5].

**Table 1.** Number of infected and percentage of infection by phylum.

Phylum /importance	Nematoda *n* (%)	Cestoda *n* (%)	Protozoa *n* (%)
Negative (0)	91; 77%	113; 97%	104; 89%
Mid infection (0 ≥ 200)	24; 21 %	2; 2%	10; 9%
Moderate infection (200 ≥ 700)	2; 2%	1; 0,5%	3; 2%
High infection (>700)	0; 0%	1; 0,5%	0; 0%
*N*	117	117	117

Where *n* represents the number of positives and *N* is the total number of animals.

**Table 2.** Parasite abundance by group and phylum, expressed as mean and standard deviation.

Abundance	G1	G2	G3	*p*
Nematoda	46.15 ± 65.24	21.54 ± 55.70	1.03 ± 6.41	0.000526
Cestoda	57.00 ± 220.01	0.00 ± 0.00	0.00 ± 0.00	0.074432
Protozoa	51.28 ± 92.66	2.05 ± 12.81	1.05 ± 8.00	0.000034

## Conclusion

In the context of this study, the parasite burdens in the animals did not result in clinical illness. The present study has certain drawbacks. Initially, adjacent human fecal samples were not analyzed, which would provide insight into whether the parasites shared by both calves and humans are the same. The study is only focused on the morphometry of the parasites. Histological or molecular techniques would verify the parasites at the species and strain levels. Standard standards have been adhered to in the processing, examination, and identification of parasites to mitigate any biases in the study.

## References

[1] Biswas H, Roy BC, Dutta PK, Hasan MM,  Parvin S,  Choudhury DK (2021). Prevalence and risk factors of *Toxocara vitulorum* infection in buffalo calves in coastal, northeastern and northwestern regions of Bangladesh. Vet Parasitol Reg Stud Rep.

[2] Asocioan Paraguaya de Cria de Bufalo (APACRIBU) (2021). Avanza cría de búfalos en Paraguay - Paraguay.

[3] Prada Sanmiguel GA, Plazas Caro E (2010). Curvas de eliminación de huevos por gramo de materia fecal de parásitos gastrointestinales en Búfalos de agua (*Bubalus bubalis*) del Magdalena Medio Colombiano. Rev Med Vet (Bogota).

[4] Uzcátegui D, Angulo-Cubillán F, Gil M (2014). Prevalencia de *Moniezia* spp. en Búfalos del municipio Colón, estado Zulia-Venezuela. Revista Cientifica de la Facultad de Ciencias Veterinarias de la Universidad del Zulia.

[5] Gorsich EE, Ezenwa VO, Jolles AE (2014). Nematode-coccidia parasite co-infections in African buffalo: epidemiology and associations with host condition and pregnancy. Int J Parasitol Parasites Wildl.

[6] Angulo RA, Noguera RR, Berdugo JA (2005). The water buffalo (*Bubalus bubalis*): an efficient user of nutrients; aspects of fermentation and ruminal digestion. Livest Res Rural Dev.

[7] Charlier J, Thamsborg SM, Bartley DJ, Skuce PJ, Kenyon F, Geurden T (2018). Mind the gaps in research on the control of gastrointestinal nematodes of farmed ruminants and pigs. Transbound Emerg Dis.

[8] Nanda AS, Brar PS, Prabhakar S (2003). Enhancing reproductive performance in dairy buffalo: major constraints and achievements. Reprod Suppl.

[9] Coman S, Berean DI, Cimpean R, Ciupe S, Coman I, Bogdan LM (2024). Clinical modalities for enhancing reproductive efficiency in buffaloes: a review and practical aspects for veterinary practitioners. Animals (Basel).

[10] Charlier J, Höglund J, Morgan ER, Geldhof P, Vercruysse J, Claerebout E (2020). Biology and epidemiology of gastrointestinal nematodes in cattle. Vet Clin North Am Food Anim Pract.

[11] Khan ZZU, Khan S, Ahmad N, Raziq A (2007). Investigation of mortality incidence and managemental practices in buffalo calves at commercial dairy farms in Peshawar City. J Agric Biol Sci.

[12] Love S, Hutchinson G (2007). WormTest for livestock and guide to egg counts. New South Wales Department of Primary Industries, Canberra, Australia.

[13] Fontenot DK, Miller JE (2012). Alternatives for gastrointestinal parasite control in exotic ruminants. Fowler’s Zoo Wild Anim Med: Curr Ther..

[14] Lejeune M, Mann S, White H, Maguire D, Hazard J, Young R (2023). Evaluation of fecal egg count tests for effective control of equine intestinal strongyles. Pathogens.

[15] Van Wyk JA, Cabaret J, Michael LM (2004). Morphological identification of nematode larvae of small ruminants and cattle simplified. Vet Parasitol.

[16] Hastutiek P, Lastuti NDR, Suwanti LT, Sunarso A,  Suprihati E,  Kurniawati DA (2022). Coproparasitological examinations and molecular determination of *Eimeria* species in Madura cattle reared on Madura Island, Indonesia. Parasitol Int.

[17] Gelaye W, Williams NA, Kepha S, Junior AM, Fleitas PE, Marti-Soler H (2021). Performance evaluation of Baermann techniques: the quest for developing a microscopy reference standard for the diagnosis of Strongyloides stercoralis. Plos Negl Trop Dis.

[18] Pinilla JC, Flórez P, Sierra M (2018). Prevalence of gastrointestinal parasitism in bovines of cesar state, Colombia. Rev Invest Vet Peru.

[19] Idris OA, Wintola OA, Afolayan AJ (2019). Helminthiases; prevalence, transmission, host-parasite interactions, resistance to common synthetic drugs and treatment. Heliyon.

[20] Tesfaye T (2021). Prevalence, species composition, and associated risk factors of small ruminant gastrointestinal nematodes in South Omo zone, South-western Ethiopia. J Adv Vet Anim Res.

[21] Quijada J, Bethencourt A, Rosales N, Vivas I, Salcedo P (2008). Prevalencia, distribución y abundancia de huevos de estróngilos digestivos y ooquistes de *Eimeria* spp en caprinos estabulados infectados naturalmente. Zootecnia Trop.

[22] Van Aken D, Dargantes A, Valdez L, Flores A, Dorny P, Vercruysse J (2000). Comparative study of strongyle infections of cattle and buffaloes in Mindanao, the Philippines. Vet Parasitol.

[23] Herd RP, Queen WG, Majewski GA (1992). Sex-related susceptibility of bulls to gastrointestinal parasites. Vet Parasitol.

[24] Jolles AE (2007). Population biology of African buffalo (*Syncerus caffer*) at Hluhluwe-iMfolozi Park, South Africa. Afr J Ecol.

[25] Sabey KA, Song SJ, Jolles A, Knight R, Ezenwa VO (2021). Coinfection and infection duration shape how pathogens affect the African buffalo gut microbiota. ISME J.

[26] Jolles AE, Ezenwa VO, Etienne RS, Turner WC, Olff H (2008). Interactions between macroparasites and microparasites drive infection patterns in free-ranging African buffalo. Ecology.

[27] Ahmed SA, Penhale WJ, Talal N (1985). Sex hormones, immune responses, and autoimmune diseases. Mechanisms of sex hormone action. Am J Pathol.

